# Predicting “Heart Age” Using Electrocardiography

**DOI:** 10.3390/jpm4010065

**Published:** 2014-03-07

**Authors:** Robyn L. Ball, Alan H. Feiveson, Todd T. Schlegel, Vito Stare, Alan R. Dabney

**Affiliations:** 1 The Jackson Laboratory, 600 Main Street, Bar Harbor, ME 04609, USA; 2 Human Adaptation and Countermeasures Division, NASA Johnson Space Center, Houston, TX 77058, USA; E-Mails: alan.h.feiveson@nasa.gov (A.H.F.); ttschlegel@gmail.com (T.T.S.); 3 Institute of Physiology, School of Medicine, University of Ljubljana, 1000 Ljubljana, Slovenia; E-Mail: vito.starc@mf.uni-lj.si; 4 Department of Statistics, Texas A&M University, 3143 TAMU, College Station, TX 77843, USA; adabney@stat.tamu.edu

**Keywords:** cardiology, personalized medicine, electrocardiogram, heart age, Bayesian statistics

## Abstract

Knowledge of a patient's cardiac age, or “heart age”, could prove useful to both patients and physicians for better encouraging lifestyle changes potentially beneficial for cardiovascular health. This may be particularly true for patients who exhibit symptoms but who test negative for cardiac pathology. We developed a statistical model, using a Bayesian approach, that predicts an individual's heart age based on his/her electrocardiogram (ECG). The model is tailored to healthy individuals, with no known risk factors, who are at least 20 years old and for whom a resting ∼5 min 12-lead ECG has been obtained. We evaluated the model using a database of ECGs from 776 such individuals. Secondarily, we also applied the model to other groups of individuals who had received 5-min ECGs, including 221 with risk factors for cardiac disease, 441 with overt cardiac disease diagnosed by clinical imaging tests, and a smaller group of highly endurance-trained athletes. Model-related heart age predictions in healthy non-athletes tended to center around body age, whereas about three-fourths of the subjects with risk factors and nearly all patients with proven heart diseases had higher predicted heart ages than true body ages. The model also predicted somewhat higher heart ages than body ages in a majority of highly endurance-trained athletes, potentially consistent with possible fibrotic or other anomalies recently noted in such individuals.

## 1. Introduction

Starc *et al.* [1] recently developed a multilinear regression model that used electrocardiographic (ECG) and other outputs such as body mass index to estimate the cardiac ages, or “heart ages”, of healthy individuals. Such estimates might be useful to both physicians and patients for better encouraging lifestyle changes that may be beneficial for cardiovascular health. Consider for example the case of a middle-aged individual with no known cardiac risk factors and a normal conventional 12-lead ECG and physical exam who is merely told that he appears to be “healthy” from a cardiovascular standpoint, *i.e.*, when being cared for by a physician under a typical “population based” clinical approach. While from the perspective of population averages this patient may be considered healthy, it would be of greater use to have a more detailed and relative measure of the individual's cardiac health. The goal of this study was to provide such a measure based on the individual's electrocardiogram (ECG).

Currently, there are several “heart age”-type prediction tools available online [2,3,4], with their predictions typically based on the answers to a series of questions regarding the subject's age, weight, health history, cholesterol level, blood pressure, *etc.* While some of these questionnaires attempt to take advantage of the known utility that Framingham risk scoring has for predicting future cardiac event risk on a population level [5], they are principally indirect and inferential tests that do not take any direct physiological information from the heart into consideration. Herein, we propose a potentially more straightforward and personalized approach that uses an extensive suite of advanced and conventional electrocardiographic measurements to more directly estimate a subject's “heart age”. Our current approach was designed to further extend the recent work of Starc *et al.*, especially from a statistical modeling viewpoint.

The limitations of strictly conventional 12-lead ECG have been well-documented [6,7,8]. However, more advanced techniques have been developed that, especially when used in combination, improve the diagnostic power of the ECG [9,10,11]. Until this time, the specific strategy of diagnosis used by Schlegel *et al.* [9,10,11] has employed a linear combination of outputs estimated by logistic regression or discriminant analysis models to determine disease status. Given this background, the principal goal of the present study was to predict a subject's heart age based on ECG outputs only after a determination was first made (clinically and by advanced ECG) that disease status was “negative”, *i.e.*, that the subject was “healthy” from the perspectives of both population-based cardiovascular medicine and advanced ECG. The question was thus: “Given an individual who is healthy and without any traditional cardiovascular risk factors other than (potentially) age itself, and given the individual's ECG outputs, what is his/her heart age (ECG age)?” The problem is that we do not observe heart age; we only observe the ECG outputs and chronological age. Thus, we cannot utilize a simple regression-type model to predict heart age. Instead we must find some means of inferring heart age based on the observables and prior information. A Bayesian approach is a natural solution.

## 2. Statistical Method

In the Bayesian paradigm, we first assume knowledge of a “prior” distribution on a parameter of interest, we then collect data to modify this prior distribution into a “posterior” distribution, and then we usually take the mean or the mode of the posterior distribution to be the point estimate of the parameter. In this case, the parameter of interest is the subject's heart age and the point estimate is the mean of the posterior distribution of the subject's heart age.

We assume that without other information, a healthy subject's heart age (*a*) is approximately normally distributed within 15 years of the subject's body age (*x*) so the prior distribution of heart age, *p*(*a*∣*x*) = *N*(*x*, 7.5^2^). The choice of *σ_a_* = 7.5 is consistent with the work of Grundy [12], who considered the use of effective age based on imaging measures of coronary artery calcium (CAC) instead of body age as input to the Framingham risk model [5]. In [12], Grundy provides a table that directly relates body age to an adjustment in Framingham risk points depending on the percentile of measured CAC for a subject's age group. From this table, average adjustments are about 4.5 risk points up or down for “greater than the 75th” and “less than the 25th” percentile CAC scores, respectively. Using the approximation that a unit increase in Framingham risk points is equivalent to about 2 years of age, one would conclude that Grundy's model would adjust age by about ±9 years at these extremes. Under the assumption that “greater than the 75th percentile” roughly corresponds to the 87.5th percentile and “less than the 25th percentile” roughly corresponds to the 12.5th percentile, then with a normal distribution, the standard deviation of adjusted age would be about 7.8 years, about the same as our assumed 7.5 years.

For a given subject, let

$$\begin{array}{lll} x & = & \text{body age}\\ y & = & \text{vector of}\;k\;\text{ECG outputs}\\ a & = & \text{heart age} \end{array}$$

By Bayes' Rule, the posterior distribution for heart age is:

(1) $$p(a|x,y)=\frac{p(a|x)p(y|x,a)}{\int p(a|x)p(y|x,a)da}$$

and the predicted heart age, *â* = *E*(*a*∣*x*, ***y***) = ∫ *ap*(*a*∣*x*, ***y***)*da*, is the estimated mean of the posterior distribution.

Specifying the distribution of the vector of ECG outputs (***y***) given heart age (*a*) and body age (*x*) is somewhat more challenging. Before including heart age (*a*) in the distribution, consider the distribution *p*(***y***∣*x*) when ***y*** is one dimensional (***y*** = *y* and *k* = 1). As discussed previously, we do not have measurements of heart age so we cannot perform a simple regression to predict heart age. However, we do have measurements of body age, and we can use that information to help estimate the distribution of the ECG outputs (*p*(***y***∣*x*)).

Assume for the moment that we have data consisting of *n* observations of *y* and *x*, say [(*y_i_*, *x_i_*)] and that we wish to build a regression model for *y_i_* conditional only on *x_i_* so that the mean of *y_i_* depends only upon *x_i_*. Examination of plots of body age *versus* some of the more important ECG outputs revealed slight non-linear trends so we assumed the following quadratic regression model:

(2) $$y_{i}|x_{i}=\beta_{0}+\beta_{1}x_{i}+\beta_{2}x_{i}^{2}+\nu_{i};\nu_{i}\sim N(0,\sigma_{\nu }^{2})$$

for *i* = 1, 2, …, *n*. In vector notation, this model can be written $\vec{y}|X\sim N(X\beta ,\sigma_{\nu }^{2}I)$, where $\vec{y}$ = (*y*_1_, *y*_2_, …, *y_n_*)*^T^*, and where

$$X=\left[\begin{array}{ccc} 1 & x_{1} & x_{1}^{2}\\ 1 & x_{2} & x_{2}^{2}\\ \vdots & \vdots & \vdots \\ 1 & x_{n} & x_{n}^{2} \end{array}\right]$$

Provided that the quadratic model holds, the Gauss-Markov Theorem ensures that we obtain an unbiased estimate of ***β*** with ordinary least squares. Therefore, ***$\hat{\beta }$*** = (***X****^T^****X***)^−1^***X****^T^$\vec{y}$* is the best linear unbiased estimator. It follows that an unbiased estimate of $\sigma_{\nu }^{2}$, is ${\hat{\sigma }}_{\nu }^{2}=\frac{1}{n-3}\sum_{i=1}^{n}e_{i}^{2}$, where ***e*** = (*e*_1_, *e*_2_,…, *e_n_*)*^T^* = *$\vec{y}$* − ***X $\hat{\beta }$*** [13].

**Figure 1:**
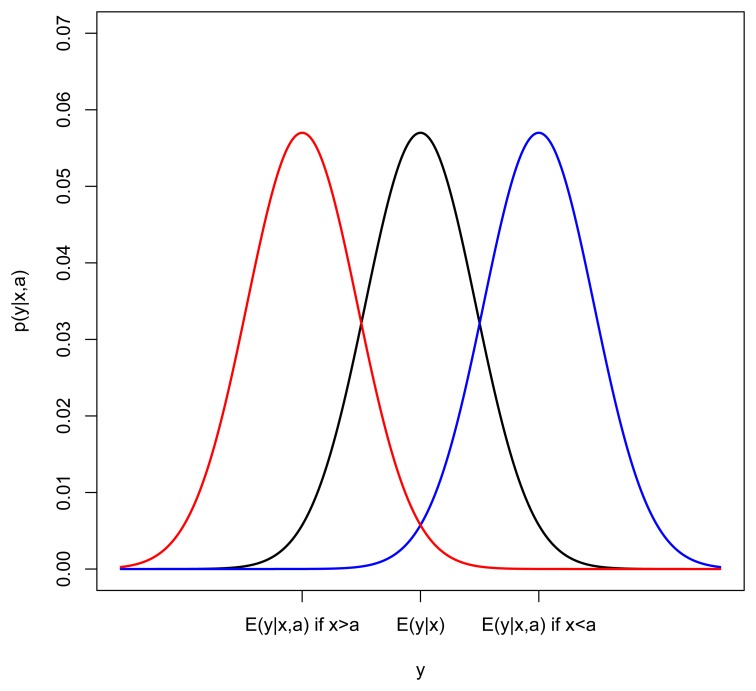
Illustration of the effect of heart age on the mean of *y*.

Given the estimates of ***β*** and $\sigma_{\nu }^{2}$, we now incorporate heart age into the model. The influence of heart age (*a*) on the distribution of the ECG variable (*y*) is best illustrated with an example. Consider the case where the ECG output increases as body age and heart age increase (*y* increases as *x* and *a* increase) and suppose we look closely at subjects with a particular body age (keep *x* fixed). If heart age (*a*) were not in the model, we would expect to see a normal distribution of *y* around E(*y*∣*x*) = *β*_0_ + *β*_1_*x* + *β*_2_*x*^2^. In Figure 1, this distribution is represented by a black curve. In the case where heart age is greater than body age, we would expect the mean of *y* to be shifted right (blue) and if heart age is less than body age, we would expect the mean of *y* to be shifted left (red). This suggests the following model for *y_i_* conditional on both *x_i_* and *a_i_*:

(3) $$(y_{i}|x_{i},a_{i})=\beta_{0}+\beta_{1}x_{i}+\beta_{2}x_{i}^{2}+\theta (a_{i}-x_{i})+\epsilon_{i}$$

where given *a_i_* and *x_i_* the residual error *ϵ_i_* ∼ *N*(0, *λ*^2^).

However, since we do not observe heart age, age, *a_i_*, how do we estimate *θ*? Comparing the body-age-only model (2) with the model that incorporates body age and heart age (3), it can be seen that *ν_i_* = *θ*(*a* − *x_i_*) + *ϵ_i_*, so that $\text{Var}\{\nu_{i}\}=\text{Var}\{\theta (a_{i}-x_{i})+\epsilon_{i}\}=\theta^{2}\sigma_{a}^{2}+\text{Var}(\epsilon_{i})$ when *a_i_* is independent of *ϵ_i_*. Recall that we have already assumed $\sigma_{a}^{2}=7.5^{2}$ and that we could estimate Var [*ν_i_*] from the residuals of the body-age-only model (2). If we could also estimate Var(*ϵ*) = *λ*^2^, we could solve for $\theta =\sqrt{\frac{\sigma_{\nu }^{2}-\lambda^{2}}{\sigma_{a}^{2}}}$, but we must take the sign of *θ* into account. Notice that if *y* and *x* have a positive increasing relationship, *θ* should be positive but, if *y* decreases as *x* increases, *θ* should be negative. This is evident by looking at the sign of *β*_1_, therefore,

(4) $$\theta =\mathrm{sgn}(\beta_{1})\sqrt{\frac{\sigma_{\nu }^{2}-\lambda^{2}}{\sigma_{a}^{2}}}$$

In order to estimate *λ*^2^ and thus, solve for *θ̂* in Equation (6), we utilized data from a previous study by Batdorf *et al.* [14] on the reproducibility and reliability of certain ECG-related outputs, which was completed by members of our research group at Johnson Space Center in 2006. In this study, there were two repeated ECG measures of *y*, taken a month apart, on *m* = 15 asymptomatic subjects (8 males and 7 females). We assumed that every subject had a fixed heart age which was not likely to change in one month, thus, any variability we observed in *y* for the subject was due to Var(*ϵ*) = *λ*^2^. Thus, we used the repeated measures to estimate Var(*y*∣*x*, *a*) = *λ*^2^ from the differences *d* between the two measurements, noting that E(*d*^2^) *=* 2*λ*^2^.

(5) $${\hat{\lambda }}^{2}=\frac{1}{2m}\sum d^{2}$$

Substituting estimates for actual parameter values in Equation (4), we were thus able to estimate *θ* by

(6) $$\hat{\theta }=\mathrm{sgn}({\hat{\beta }}_{1})\sqrt{\frac{{\hat{\sigma }}_{\nu }^{2}-{\hat{\lambda }}^{2}}{7.5^{2}}}$$

After substituting a *N*(*x*, 7.5^2^) density for *p*(*a*∣*x*) and the estimated normal density of (*y_i_*∣*x_i_*, *a_i_*) corresponding to the model (3) into the expression for *p*(***y***∣*x*, *a*) in Equation (1) it can be shown that (see [15], pp. 43–45) the posterior for a subject's heart age is a Normal distribution with an estimated mean equal to:

(7) $$\hat{a}=x+\left\{\frac{\hat{\theta }(y-\hat{\beta }\underline{x})}{{\hat{\lambda }}^{2}}\right\}{\left\{\frac{1}{\sigma_{a}^{2}}+\frac{{\hat{\theta }}^{2}}{{\hat{\lambda }}^{2}}\right\}}^{-1}$$

where $\sigma_{a}^{2}=7.5^{2}$ and *x* = [1, *x*, *x*^2^]*^T^*.

Recall, the above formulation is for a single ECG output. To generalize for *k* ECG outputs, it can be shown that the predicted heart age for the subject with body age *x* and vector of *k* ECG outputs ***y*** is:

(8) $$\hat{a}=x+\{\theta^{T}\Lambda^{-1}(y-\beta \underline{x})\}{\left\{\frac{1}{\sigma_{a}^{2}}+\theta^{T}\Lambda^{-1}\theta \right\}}^{-1}$$

Here, ***y*** = [*y*_1_, *y*_2_, …, *y_k_*]*^T^* is now a vector of the *k* ECG outputs, ***θ*** = [*θ*_1_, *θ*_2_,…, *θ_k_*]*^T^* is a vector of *k* different values of *θ* in Equation (3), ***β*** is a *k* × 3 matrix of regression coefficients satisfying *E*(***y***∣*x*) = ***β****x*, and Λ is the *k* × *k* covariance matrix of the *k*-variate analog of *ϵ_i_* in Equation (3). To implement Equation (8) in practice, one can estimate ***β*** by multiple regression and **Λ** by the multivariate analog of Equation (5). A relatively simple way of estimating the *j*-th component of ***θ*** is to apply Equation (6) for the *j*-th component of (*y*) where *λ̂* is replaced by *λ̂_jj_*. An example is given in Section 5. Please see [15], pp. 45–48, for the mathematical details.

## 3. Demonstration Data

To demonstrate our method for predicting heart age, we used a database of de-identified advanced 12-lead ECG recordings from healthy individuals with no known cardiac risk factors other than (potentially) age itself. Secondarily, mainly just to explore the method's initial performance in other groups, we then also applied the method to groups of individuals with risk factors, with proven heart disease, or with a high level of endurance athletic training. All recordings were obtained under previous Institutional Review Board (IRB) approvals or under updated IRB exemptions for previously collected and de-identified clinical data. The included subjects consisted of healthy and diseased volunteers from the Johnson Space Center and partner hospitals, for example from the University of Ljubljana hospitals and clinics in Ljubljana, Slovenia, and elsewhere [11].

Each subject was classified according to their cardiac disease status and, if disease was present, then also to the form and severity of disease. Disease status was diagnosed based on results from clinical imaging tests (current “gold standards”) so that if a subject had heart disease, the form and severity of heart disease was generally known [11]. Subjects were classified as “healthy” if they had no cardiovascular or other systemic disease and also did not have other risk factors, such as hypertension, smoking, or diabetes [11].

From this dataset, we identified 1,438 subjects who were at least twenty years old, from whom a resting ∼5-min 12-lead ECG test had been obtained after written informed consent, and who were categorized as healthy, diseased, or as having risk factors. Some of the subjects were endurance athletes, and others had risk factors such as diabetes or high blood pressure and were not ultimately assigned a definitive diagnosis because cardiac imaging was not specifically indicated. The subjects were thus classified into 4 groups: healthy non-athletes (HNA); healthy athletes (ATH); subjects with risk factors and no diagnosis (RFS); and subjects who had known disease by cardiac imaging (DIS). Originally, there were 728 subjects in the HNA group; these were further subdivided into a training set (*N* = 545) for estimating $\sigma_{\nu }^{2}$, ***β***, and ***θ*** and a test set (*N* = 183) for validation of heart age predictions. Subjects in the test set were chosen by a 25% systematic sample after ordering on age. We also made predictions of heart age for the other groups to examine the effect of disease and risk factors on predicted heart age. The athletes were asymptomatic volunteers of both genders who had no evidence of cardiac disease based on a negative history and physical examination. They were all endurance-trained athletes, and a majority also had cardiac magnetic resonance imaging scans demonstrating no evidence of hypertrophic cardiomyopathy nor any other gross clinical pathology [10]. Descriptive statistics for all groups are given in Table 1.

**Table 1:** Descriptive statistics for healthy non-athletes (HNA), healthy endurance-trained athletes (ATH), subjects with risk factors (RFS), and subjects with disease (DIS).

**Group**	**#Subjects**	**%Females**	**%20–40**	**%41–60**	**%60 and older**
HNA(train)	545	41%	56%	36%	8%
HNA(test)	183	43%	55%	36%	9%
ATH	48	38%	92%	6%	2%
RFS	221	47%	10%	59%	31%
DIS	441	34%	7%	50%	43%

## 4. Gender Effects

Experts agree that women's hearts age differently than men's hearts [16,17,18]. Assuming that cardiac aging in asymptomatic individuals can be construed as tantamount to the gradual acquisition of coronary atherosclerotic plaque with time, then on average, the hearts of women tend to age more slowly than those of men until after the female menopause, at which time cardiovascular aging in women quickly catches up to that of men. As a result, we computed all predictions of heart age with gender-specific estimates of $\sigma_{\nu }^{2}$, ***β***, and ***θ***. However, with the small amount of repeatability data available for estimating **Λ**, we had to assume **Λ** was the same for both genders. Of course, if more data were available for both genders, there is no reason why **Λ** could not also be estimated separately. We checked for a gender effect by comparing the predicted heart ages from separate gender-specific (GS) models and a model with the same ECG variables that does not take gender into account (NGS). We found that if we do not take gender into account, the NGS model is, on average, biased upwards for males; 95% confidence for mean (NGS – GS) = (0.86 years, 0.99 years) and biased downwards for females; 95% confidence for mean (NGS – GS) = (−1.48 years, −1.22 years). The associated *t*-tests were significant (*p*-value < 0.0001 for both comparisons). Based on these results and also from the results of a comparison of the GS and NGS models by use of the Bayes Factor [19], we made the decision to report gender-specific parameter estimates in the sections that follow.

## 5. Model Estimation

To decide which ECG outputs should be included in the model, we drew on the results from [11]. The parameter estimates shown below, in Equation (10), were obtained from two ECG-derived outputs *y*_1_ and *y*_2_ (*k* = 2). The first ECG-derived output, *y*_1_, varies with age and disease and *y*_2_ varies with disease. To arrive at these linear combinations (*y*_1_ and *y*_2_), we performed a logistic regression with *ν*_1_, *ν*_2_ as the explanatory variables and the response for healthy non-athlete group = 0 and the response for subjects with heart disease = 1. We then extracted the coefficients (*γ*_1_, *γ*_2_) from the two logistic regression equations.

$$y_{1}={\gamma_{1}}^{T}\upsilon_{1}\text{and}y_{2}={\gamma_{2}}^{T}\upsilon_{2};y={\left[y_{1},y_{2}\right]}^{T}$$

(9) $$\upsilon_{1}=\left[\begin{array}{c} 1\\ \text{Taxis}\\ \text{Pd}\\ \sin(\text{FrQRSMax}*\pi /180)\\ \log(\text{HFP})\\ \log(\text{RMSsum})\\ \log(\text{SpatialJT}) \end{array}\right],\upsilon_{1}=\left[\begin{array}{c} 1\\ \text{IIQTVI}\\ \text{UnExQTVI}\\ \sin(\text{QRSaxis}*\pi /180)\\ \text{Pd}\\ \text{MeanQRS}-T\\ \log(\text{IDR}) \end{array}\right]$$

(10) $$\gamma_{1}=\left[\begin{array}{c} -33.0669357\\ -0.007471284\\ 0.0524961\\ -3.977162174\\ -0.75406667\\ 0.295048301\\ 5.607131563 \end{array}\right]\text{and}\gamma_{2}=\left[\begin{array}{c} -5.561987914\\ 3.278798871\\ 1.482313958\\ -2.6315664\\ 0.090181799\\ 0.048045487\\ 1.426993361 \end{array}\right]$$

In Equation (9), Taxis is the axis of the T wave in the conventional ECG frontal plane; Pd is the P-wave duration on the conventional ECG; FrQRSMax is the axis of the QRS loop in the derived vectorcardiographic frontal plane after transformation of the conventional ECG to the Frank X-Y-Z leads through use of Kors *et al.*'s regression transform [20]; HFP is the high frequency power of beat-to-beat RR interval variability as measured through the Lomb periodogram technique [11]; RMSsum is the sum of the high frequency QRS root mean squared voltages across all 12 signal-averaged leads after bandpass filtering between 150–250 Hz [11]; SpatialJT is the JT interval as measured “spatially” from the vector magnitude of the derived vectorcardiographic (Frank X-Y-Z) leads; IIQTVI is the so-called QT variability index in lead II and UnExQTVI is the “index of unexpected QT variability” in lead V5 [11,21]; QRSaxis is the axis of the QRS wave in the conventional ECG frontal plane; MeanQRS-T is the spatial mean QRS-T angle as obtained from the derived vectorcardiogram [11]; and IDR is the “intradipolar ratio” of T-wave complexity as derived from singular value decomposition and signal averaging of the T wave [11]. A more detailed description of most of the above ECG-derived outputs can be found in Supplemental Table 1 of the supplementary material corresponding to [11].

The resulting parameter estimates for the gender-specific model (1 corresponds to males, 2 corresponds to females) were:

$$\begin{array}{lll} {\hat{\theta }}_{1} & = & {\left[0.170889,0.265498\right]}^{T}\\ {\hat{\theta }}_{2} & = & {\left[0.1428490.245966\right]}^{T}\\ {\hat{\beta }}_{1} & = & \left[\begin{array}{lll} -5.951124 & 0.133771 & -0.000538\\ -10.382251 & 0.210635 & -0.001508 \end{array}\right]\\ {\hat{\beta }}_{2} & = & \left[\begin{array}{lll} -4.880738 & 0.072246 & -0.00052\\ -7.993616 & 0.092820 & -0.00220 \end{array}\right]\;\text{and}\\ {\hat{\Lambda }}^{-1} & = & \left[\begin{array}{ll} 4.649546 & -0.064033\\ -0.064033 & 0.519129 \end{array}\right] \end{array}$$

For a given subject with

$$\begin{array}{lll} j & = & 1\text{if male and}j=2\text{if female}\\ x & = & \text{body age}\\ \underline{x} & = & {\left[1,x,x^{2}\right]}^{2}\\ y & = & {\left[y_{1},y_{1}\right]}^{T}\text{where}y_{1}={\gamma_{1}}^{T}\upsilon_{1},y_{2}={\gamma_{2}}^{T}\upsilon_{2} \end{array}$$

the predicted heart age for the subject is

(11) $$\hat{a}=x+\left\{{\hat{\theta }}_{j}^{T}{\hat{\Lambda }}^{-1}\left(y-{\hat{\beta }}_{j}\underline{x}\right)\right\}{\left\{\frac{1}{\sigma_{a}^{2}}{\hat{\theta }}_{j}^{T}{\hat{\Lambda }}^{-1}{\hat{\theta }}_{j}\right\}}^{-1}.$$

## 6. Results

**Figure 2:**
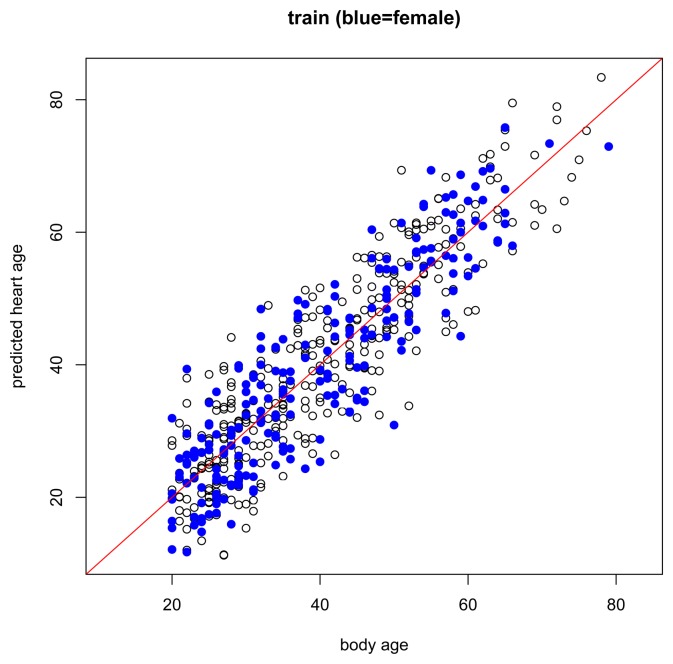
Body age *versus* predicted heart age in the training set. Black circle = male. Blue dot = female.

**Figure 3:**
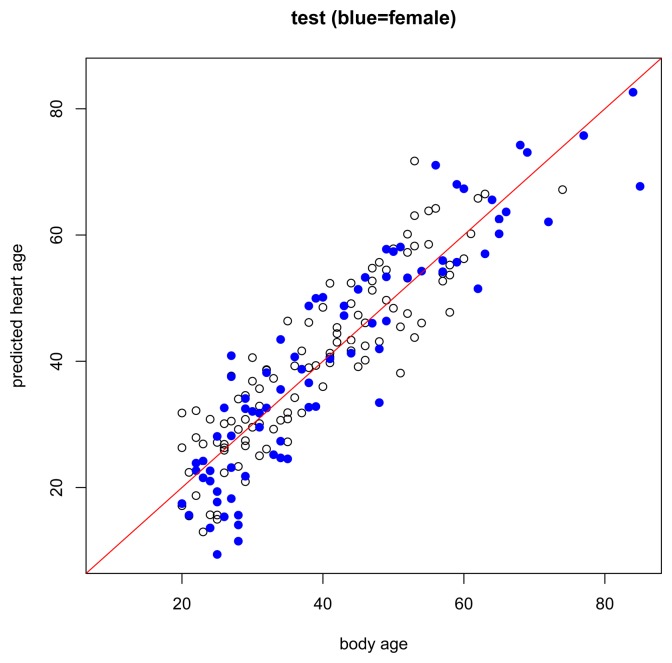
Body age versus predicted heart age in the test set. Black circle = male. Blue dot = female.

The results of the main gender-specific model are shown in Figure 2 and Figure 3 below. Normally, when making predictions, we want the predicted values to be equal to the observed values. In the figures below, this is symbolized by heart age equals body age (red line) where the *x*-axis is the subject's body age and the *y*-axis is the subject's predicted heart age. If the subject's body age equals the subject's predicted heart age, it will fall on the red line. If the subject's predicted heart age is higher than the subject's body age, it will be above the red line, and if the subject's predicted heart age is lower than the subject's body age, it will be below the red line. In this case, we want predicted heart age to be centered around the subject's body age, but not necessarily equal to the subject's body age because we want to take the subject's ECG into account when computing heart age. In the training data, we see this for both females and males (Figure 2). Predicted heart ages are centered around the red line with some variability according to each subject's ECG. We also observe this phenomenon in the test set, Figure 3.

**Figure 4:**
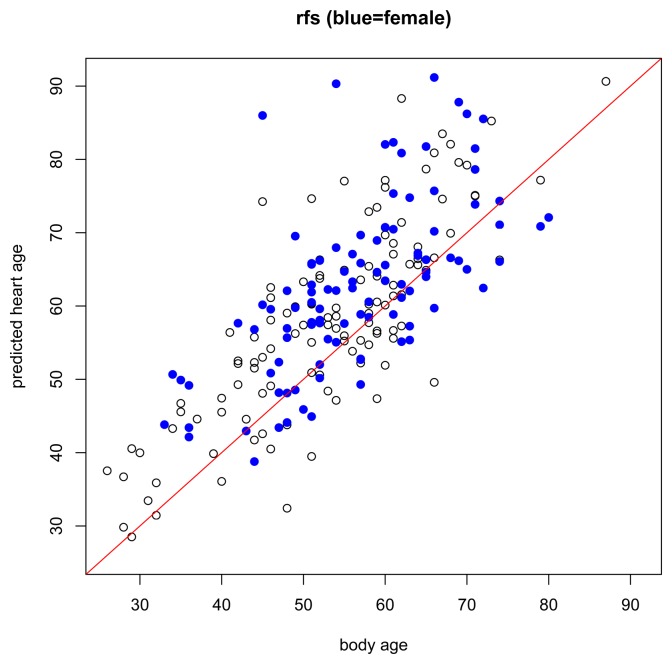
Body age *versus* predicted heart age for subjects with risk factors. Black circle = male. Blue dot = female.

**Figure 5:**
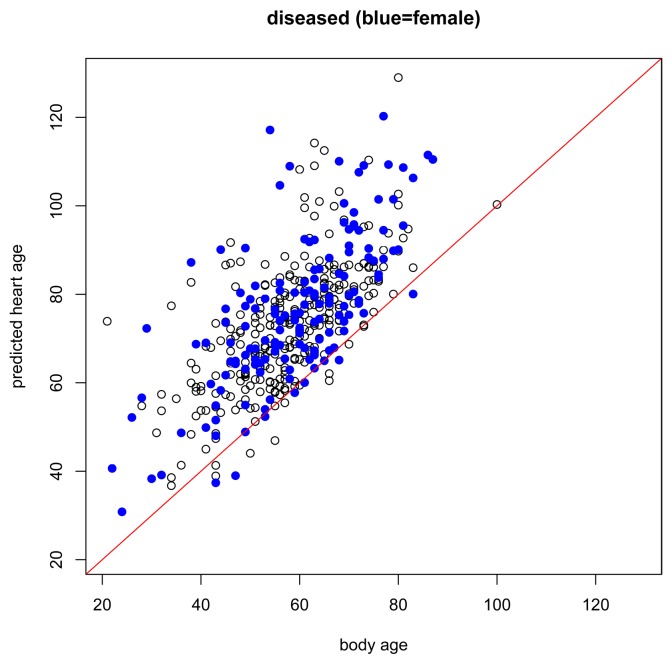
Body age *versus* predicted heart age for subjects with disease. Black circle = male. Blue dot = female.

While the assumptions of the model are based on a healthy non-athlete population, it is also interesting to preliminarily explore how the model performs under other conditions, for example, in subjects with risk factors or known heart disease, or in endurance-trained athletes. In subjects with risk factors (Figure 4), we expect that most will have higher predicted heart ages than their respective body ages but this should not be the case for everyone. Just because a person has a risk factor does not mean that he/she also has heart disease. And, in subjects with heart disease (Figure 5), we expect most subjects to have a higher predicted heart age than their body age. Indeed, these results bear this out.

**Figure 6:**
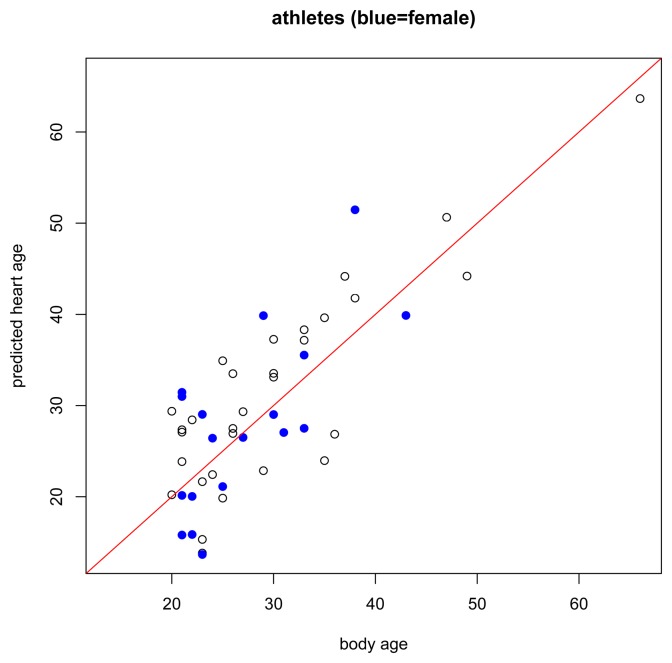
Body age *versus* predicted heart age for athletes. Black circle = male. Blue dot = female.

Regarding the healthy athlete group (Figure 6), interestingly, we did not find that athletes had lower predicted heart ages than their body age. In fact, 56.25% of the athletes had a predicted heart age higher than their body age whereas 51% of subjects in the healthy non-athlete training set had a predicted heart age higher than their body age. Recall that these athletes are endurance-trained elite athletes. A recent Mayo Clinic publication [22] found that intense endurance athletic activity may often induce subtle fibrotic and other cardiac damage through excessive training. Our predictions appear to support this hypothesis.

## 7. Discussion and Conclusions

Our heart age model was designed for healthy individuals at least 20 years old. Thus, it should be considered for use only in such individuals, wherein the predicted heart age is centered around body age and yet takes the variability of each subject's ECG into account. It was, however, additionally interesting just to briefly examine what the model revealed for other groups, including those with risk factors or with proven heart disease, and for highly endurance–trained athletes. About three-fourths of the subjects with risk factors, almost all of the subjects with proven heart disease, and 56.25% of the athletes had higher predicted heart ages than body ages. Regarding the athletes, our ECG-based model therefore potentially supports recent findings showing relatively more cardiac fibrosis and coronary calcifications in such individuals [22], changes that historically have more frequently been construed as signs of aging [23,24].

Given the lack of any true gold standard for “heart age”, we did not attempt to compare the veracity of our ECG-based heart age predictions to that of online calculators or other methods that utilize less direct inputs similar to those used in the more traditional Framingham risk score calculators. Such comparisons must await future studies, ideally studies wherein an acceptable gold standard result for “heart age” has also been simultaneously obtained.

We believe our model can provide patients and their physicians with potentially useful additional personalized cardiac health information that, in sufficiently motivated patients, might potentially lead to earlier institution of heart-healthier lifestyles. For example, for any patient who has not been diagnosed with heart disease, but who nonetheless has a higher predicted heart age than chronological body age, the overt demonstration of the relatively high heart age by a simple and “heart-direct” technique like ECG might sufficiently motivate many such patients to institute better dietary, exercise, sleep and other habits.
